# Attitudes Toward Psychotherapeutic Treatment and Health Literacy in a Large Sample of the General Population in Germany: Cross-Sectional Study

**DOI:** 10.2196/67078

**Published:** 2025-01-24

**Authors:** Rebekka Schröder, Tim Hamer, Ralf Suhr, Lars König

**Affiliations:** 1 Stiftung Gesundheitswissen Berlin Germany

**Keywords:** health literacy, mental health literacy, attitude to health, cross-sectional studies, Germany, adults, representative, psychotherapy

## Abstract

**Background:**

Prevalences of mental disorders are increasing worldwide. However, many people with mental health problems do not receive adequate treatment. An important factor preventing individuals from seeking professional help is negative attitudes toward psychotherapeutic treatment. Although a positive shift in attitudes has been observed in recent years, there is still substantial stigma surrounding psychotherapeutic treatment. First studies have linked higher health literacy with more positive attitudes toward psychotherapy, but more research is needed in this area.

**Objective:**

This study aimed to examine how general and mental health literacy are associated with attitudes toward psychotherapeutic treatment in Germany. Additionally, associations between sociodemographic factors, experience with psychotherapy, and attitudes toward psychotherapy were explored.

**Methods:**

A random sample was drawn from a panel representative of the German-speaking population with internet access in Germany and invited to participate in the study via email. Overall, 2000 individuals aged ≥16 years completed the web-based survey with standardized questionnaires in September and October 2022. Attitudes toward psychotherapy and both general and mental health literacy were assessed using the Questionnaire on Attitudes Towards Psychotherapeutic Treatment (QAPT) with 2 subscales (“positive attitudes” and “non-acceptance of society”), the European Health Literacy Survey instrument (HLS-EU-Q16) and the Mental Health Literacy Tool for the Workplace (MHL-W-G). Associations between the questionnaire scales were assessed with Pearson correlations. Additionally, basic sociodemographic information and information on personal and family experiences with psychotherapy were collected. Pearson correlations (age), ANOVAs (level of education and subjective social status), and *t* tests (experience with psychotherapy, gender, and migration background) were used to analyze how these relate to attitudes toward psychotherapy.

**Results:**

More favorable attitudes toward psychotherapy and lower perceived societal nonacceptance were found in those with higher general (*r*=0.14, *P*<.001; *r*=−0.32, *P*<.001, respectively) and mental health literacy (*r*=0.18, *P*<.001; *r*=−0.23, *P*<.001, respectively). Participants with treatment experience for mental health problems (t_1260.12_=−10.40, *P*<.001, Cohen *d*=−0.49; t_1050.95_=3.06, *P*=.002, Cohen *d*=0.16) and who have relatives with treatment experience (t_1912.06_=−5.66, *P*<.001, Cohen *d*=−0.26; t_1926_=4.77, *P*<.001, Cohen *d*=0.22) reported more positive attitudes and higher perceived societal acceptance than those without treatment experience. In terms of sociodemographic differences, being a woman (t_1992_=−3.60, *P*<.001, Cohen *d*=−0.16), younger age (*r*=−0.11, *P*<.001), higher subjective social status (*F*_2,1991_=5.25, *P*=.005, η^2^=.005), and higher levels of education (*F*_2,1983_=22.27, *P*<.001, η^2^=.021) were associated with more positive attitudes toward psychotherapeutic treatment. Being a man (t_1994_=5.29, *P*<.001, Cohen *d*=0.24), younger age (*r*=−0.08, *P*<.001), and lower subjective social status (*F*_2,1993_=7.71, *P*<.001, η^2^=.008) were associated with higher perceived nonacceptance of psychotherapy.

**Conclusions:**

Positive associations between attitudes toward psychotherapy and both general and mental health literacy were delineated. Future studies should investigate whether targeted health literacy interventions directed at individuals with lower general and mental health literacy might also help to improve attitudes toward psychotherapeutic treatment and help-seeking behavior.

## Introduction

In Germany, almost 30% of the population are estimated to have a mental disorder [1] but only about 10% have received treatment in the past year [2]. This means that about two-thirds of those with mental health problems do not receive adequate treatment. While the number of individuals not seeking help for mental disorders has decreased in recent years, the majority of individuals with mental disorders still do not seek treatment [3]. With a global rise in the prevalence of mental disorders [4], it becomes even more important to understand the factors that influence whether an individual seeks help or not.

Psychotherapy is considered effective for treating many mental disorders [5]. However, there are several reasons that prevent individuals from seeking psychotherapy. These encompass structural challenges such as long waiting periods and underprovision, especially in rural areas [2,6,7], as well as individual reasons often related to stigma against mental disorders and psychotherapy [8,9]. Specifically, in self-reports, about one-third of respondents report negative attitudes and stigma toward psychotherapy, with more negative attitudes in male individuals and in individuals with lower education [10]. Similarly, almost a third of the respondents believed that professional care was worse than or equal to no help when faced with serious emotional problems in a large European study [8]. In recent years, attitudes toward professional help, particularly toward psychotherapeutic and psychiatric treatment, have improved both on a global level and in Germany [11,12]. In addition, perceived public stigma toward people with mental disorders has substantially decreased in the past 30 years [13]. At the same time, the individual wish for social distance toward people with depression slightly decreased but increased toward people with schizophrenia [11,14]. Furthermore, in many non-Western countries, there are still substantial negative attitudes toward individuals with mental illnesses [15,16]. Critically, it has been shown that negative attitudes toward help seeking and stigmatizing attitudes toward people with mental illness predict help-seeking behavior [7,17-20]. In summary, this means that although some progress has been made in recent years, there is still considerable stigma associated with both mental illness and its treatment, which needs to be addressed to increase the number of individuals who receive treatment in addition to reducing structural barriers.

Health literacy refers to “people’s knowledge, motivation and competencies to access, understand, appraise, and apply health information in order to make judgments and take decisions in everyday life concerning health care, disease prevention and health promotion to maintain or improve quality of life during the life course” [21]. Low health literacy has been associated with several negative health-related outcomes, including higher rates of hospitalization, lower medical treatment adherence, decreased quality of life, fewer health-promoting behaviors, and higher mortality [22-27]. Mental health literacy was developed from general health literacy and applies the concept to mental health. It is defined as “understanding how to obtain and maintain positive mental health; understanding mental disorders and their treatments; decreasing stigma related to mental disorders; and, enhancing help-seeking efficacy” [28]. High mental health literacy has a number of positive correlates such as mental well-being, physical activity, life satisfaction, healthy eating habits, and self-efficacy [29-31].

First studies have linked increased health literacy with lower stigma and more positive attitudes toward mental disorders and psychotherapy [12,19,30,32-34]. For example, Svensson and Hansson [32] have shown that a higher degree of mental health literacy is related to less stigma and a lower wish for social distance toward individuals with depression and to a lesser extent also toward individuals with schizophrenia. However, this pattern of results was not found in all studies. In a Swiss community sample of young adults, no direct association between mental health literacy and the wish for social distance was delineated. Instead, it was shown that increased mental health literacy may also lead to more stigma by enhancing stereotypes of individuals with mental disorders (eg, perceived dangerousness) due to higher endorsement of biogenetic causal explanation of mental disorders [35]. It has been demonstrated in diverse cultural contexts that specific interventions may enhance both general and mental health literacy [36-41]. Moreover, it has been shown in longitudinal studies that more positive treatment attitudes can predict more frequent mental health service use in the future [19,20]. Along with significantly reducing structural barriers, indirectly improving attitudes toward psychotherapeutic treatment via increased health literacy may therefore be an important starting point to ensure that more people with mental health problems receive professional help.

Although some studies have started to investigate the relationship between general and mental health literacy and attitudes toward psychotherapeutic treatments, there are still substantial gaps in the literature as well as some limitations of previous studies that need to be addressed in order to get a better understanding of how to increase the number of individuals seeking psychotherapeutic treatment. First, in past investigations, mental health literacy was often assessed via unlabeled vignettes [32,35] describing the symptoms of a specific disorder (eg, depression or schizophrenia), and high mental health literacy was assigned to those individuals who correctly identified these vignettes. While this covers a small aspect of the broad definition of mental health literacy, that is, the recognition of mental disorders, other important aspects, such as promoting positive mental health, are not included [28]. This limitation in previous methodological approaches is why we decided to use an instrument with a broader scope to assess mental health literacy in the present investigation [42,43]. Second, the literature on general health literacy and how it relates to treatment attitudes is relatively sparse and there is a need to replicate and extend previous findings [34]. Third, there is some evidence to suggest that there are substantial interindividual differences in attitudes toward psychotherapy in the community [19,34]. However, the specific determinants of these differences need to be explored more in depth to identify potentially vulnerable segments of the population that could particularly benefit from interventions. For example, it has been shown that gender, age, level of education, and social status play an important role in accessing psychiatric and psychotherapeutic services and might therefore also be related to attitudes [2,10]. In addition, having a migration background or being part of an ethnic minority has been associated with access to mental health care [44-48]. Finally, negative attitudes might be improved by hearing about the positive experiences of others or making these experiences themselves [19,49-51], which is why we decided to include 2 questions on personal and family experience with psychotherapy.

To address these previous limitations and gaps in the literature, in this study, we aimed to explore the relationship between attitudes toward psychotherapy and both general and mental health literacy with comprehensive questionnaires and in a large sample. Furthermore, we aimed at exploring how additional factors including basic sociodemographic factors and experience with psychotherapy relate to attitudes toward psychotherapy in order to inform the future development of target group-specific interventions with the aim of reducing individual barriers to seeking professional help.

## Methods

### Ethical Considerations

The ethics committee of the Berlin Medical Association had no ethical or professional objections to the study protocol (reference Eth-39/22). Participants gave informed consent to take part in the study. They were not directly compensated for their participation by the independent nonprofit foundation Stiftung Gesundheitswissen. Data collection was conducted by the market research institute forsa (forsa Gesellschaft für Sozialforschung und statistische Analysen mBH), which was commissioned and financially compensated for this work by the nonprofit foundation Stiftung Gesundheitswissen. Importantly, Stiftung Gesundheitswissen did not have any influence on the data collected and the direction of the results. Forsa provided only anonymized data to the Stiftung Gesundheitswissen.

### Data Acquisition

Data collection was conducted by forsa using the forsa.omninet panel. Forsa.omninet is a representative panel for the German-speaking population with internet access aged 14 years and older in Germany and currently has around 100,000 participants. Panelists are recruited exclusively offline, that is, by telephone within the framework of forsa.omniTel, a telephone omnibus survey of forsa, in which randomly selected individuals aged 14 years and older are interviewed daily. The composition of the panel is continuously monitored on the basis of key characteristics (eg, region, age, and gender) and recruitment is adjusted accordingly. In addition, approximately 1000 new individuals are recruited monthly. There is no possibility to apply for participation in the panel so the selection of the participants is solely controlled by a random sampling procedure. Inclusion criteria for this investigation were the age of 16 years or older; sufficient German knowledge; and being able to use a computer, laptop, or mobile device for the survey. The only exclusion criterion was not fulfilling the inclusion criteria. With the aim of collecting data from at least 2000 individuals, 3927 panelists aged 16 years and older were randomly drawn from the panel and invited to take part in the survey by email. Exactly 2000 participants completed the study, equaling a response rate of 50.9% (2000/3927). The invitation email contained brief information on the overall topic of the survey as well as the incentive. If necessary, the selected panelists were reminded about their participation in 2 further emails. Recruitment and data collection took place in September and October 2022 with a standardized questionnaire presented in the form of computer-assisted web interviews. The total questionnaire consisted of 155 items, of which not all are relevant to this investigation. For economic reasons, data collection for different research objectives was combined. Results are published in separate and independent publications [31,52].

To minimize the effects of nonresponse and potential bias due to systematically missing data from certain population segments, survey weights were calculated by forsa using an iterative proportional fitting approach with the following weight variables and combinations: (1) gender × age (16-29 years, 30-45 years, 46-64 years, or ≥65 years) × region (West Germany and Berlin, or East Germany) and (2) federal state. This procedure resulted in a single weighting factor for each individual applied before further statistical analyses. The weighting was informed by the population update of the German Federal Statistical Office (as of December 31, 2020).

### Measures

#### Attitudes Toward Psychotherapy

Attitudes toward psychotherapy were assessed with the German version of the Questionnaire on Attitudes Towards Psychotherapeutic Treatment (QAPT) in its revised 2-factorial form [44,53,54]. The questionnaire consists of 11 items answered on a 4-point Likert scale ranging from “do not agree” to “agree.” The items address how the respondents perceive psychotherapeutic treatments. The 2 subscales are “positive attitudes” (6 items) and “non-acceptance of society” (5 items). The positive attitude scale reflects the individuals’ beliefs about the positive effects of psychotherapeutic treatment and the competence of psychotherapists, whereas the nonacceptance scale reflects the fear of stigma and social isolation from others. The second scale is originally labeled “acceptance of society” (“Akzeptanz der Gesellschaft” in German [54]). However, as the items of this scale are negatively coded, that is, higher values indicate lower acceptance, we decided to label the scale “nonacceptance of society” for easier interpretability. For each scale, a mean value is calculated across all items. The mean value reaches from 1 to 4 with higher values indicating more positive attitudes and higher nonacceptance (eg, lower acceptance), respectively.

#### Health Literacy

General health literacy was assessed with the German translation of the short version of the European Health Literacy Survey instrument (HLS-EU-Q16 [55-57]). This 16-item questionnaire addresses the subjective difficulty in accessing, understanding, appraising, and applying information in the fields of health care, disease prevention, and health promotion. Respondents are asked to answer each item on a 4-point Likert scale with the options “very easy,” “fairly easy,” “fairly difficult,” and “very difficult.” An overall sum score is calculated for the dichotomized items (eg, 1=“fairly easy” and “very easy,” 0=“fairly difficult” and “very difficult”). The sum score ranges from 0 to 16, with higher values indicating higher health literacy. Groups of individuals with varying degrees of health literacy were determined using this sum score: inadequate health literacy (scores 0-8), problematic health literacy (scores 9-12), and adequate health literacy (scores 13-16).

#### Mental Health Literacy

Mental health literacy was assessed with the German version of the Mental Health Literacy Tool for the Workplace (MHL-W-G [42,43]). Instructions were slightly adapted so that they also apply to people who are not currently working. Specifically, participants were instructed to imagine they worked if this was not the case. The instrument presents 4 case vignettes, in each of which current symptoms and the circumstances of one individual are described. The vignettes differ in the nature of the mental health issue, its impact on workplace performance, and the gender of the individual. Importantly, the nature of the mental health issue is not explicitly labeled. For each vignette, the same 4 questions are answered covering important aspects of mental health literacy, namely the ability to recognize specific mental disorders, knowledge and beliefs about risk factors and prevention, knowledge and attitudes to facilitate help seeking, and knowledge and beliefs about mental health interventions. After reading each of the vignettes, participants are asked to rate their knowledge about what might be happening, how they could prevent the situation from worsening, what to say or do in the situation, and which resources and services might be helpful. Answers are given on a 5-point Likert scale ranging from “very low” to “very high”. A sum score is calculated by adding up the ratings of each of the 4 questions in each of the 4 vignettes (ranging from a minimum of 16 to a maximum of 80 points), with higher scores reflecting higher levels of self-reported mental health literacy. So far, no generally accepted cut-off scores have been developed to compare groups of individuals with varying levels of mental health literacy using this questionnaire, which is why we do not present stratified data here.

#### Experience with Psychotherapy

In addition, the participants were presented with 2 simple yes-or-no-questions to assess whether they have ever received any treatments for mental disorders themselves and whether any of their close family members have ever received treatment for mental disorders.

#### Sociodemographic Information

Participants provided basic sociodemographic information including gender (men, women) and age.

In addition, participants were asked to provide their highest level of formal education. Participants were then categorized into 3 groups: low, middle, and high level of education. The low level of education is equivalent to no formal education or basic secondary school (ohne Haupt-/Volksschulabschluss, Haupt-/Volksschulabschluss); the middle level of education is equivalent to intermediate secondary school (Mittlere Reife, Realschulabschluss, Fachschulreife, Abschluss der Polytechnischen Oberschule, Fachhochschulreife, Abschluss einer Fachoberschule); and the high level of education is equivalent to most advanced secondary school, for example, grammar schools to obtain a general or specialized university entrance qualification or a university degree (Abitur, allgemeine oder fachgebundene Hochschulreife, Fach-/Hochschulstudium).

Subjective social status was assessed with the German version of the MacArthur scale [58,59], in which the participants rank themselves relative to other members of society. They are presented with a metaphor, in which a ladder represents the social structure of society with the highest ladder rung representing the individuals in society with the highest status and the bottom rung presenting the individuals in society with the lowest status. The respondents are asked to identify the rung on which they place themselves. Three categories of subjective social status were determined according to the respondents’ answers, that is, low subjective social status (scores 1-4), middle subjective social status (scores 5-7), and high subjective social status (scores 8-10).

Migration status was assessed with a simple yes-or-no-question. The participants were asked to indicate if they have a migration background. They were instructed to answer with yes if they or one of their parents were not born in Germany.

### Statistical Analysis

Statistical analyses were performed with the software SPSS (version 29.0.2.0; IBM). All analyses reported in the main manuscript were conducted with the weighted dataset (details on weighting can be found in the “data acquisition” paragraph). Results for the unweighted dataset can be found in the Multimedia Appendix 1. Cronbach α was calculated to determine the internal consistency of the attitudes and nonacceptance subscales of the QAPT as well as the HLS-EU-Q16 and MHL-W-G total scores. The resulting reliability coefficients were interpreted using the following rule of thumb: >.90 excellent, >.80 good, >.70 acceptable, >.60 questionable, >.50 poor, and <.50 unacceptable [60]. For all inferential analyses, a threshold of α=.05 was used as the significance level and 2-sided *P* values are reported. Associations between the 2 attitudes toward psychotherapy scales and both mental and general health literacy were assessed with Pearson correlations, including 95% CI. Likewise, for the associations between the 2 attitudes toward psychotherapy scales and the metric sociodemographic measure of age, Pearson correlations were calculated. For each of the dichotomous sociodemographic measures and the 2 experiences with treatment for mental disorder questions (ie, gender, migration status, personal experience, and the experience of a close family member) separate 2-tailed *t* tests were performed for the 2 attitudes toward psychotherapy scales. Effect sizes for the *t* tests are reported as Cohen *d,* with the values of Cohen *d*=0.20, Cohen *d*=0.50, and Cohen *d* = 0.80 corresponding to small, medium, and large effects, respectively [61]. If the Levene test for equality of variances indicated that variances were not homogeneous, degrees of freedom were adjusted accordingly. For each categorial sociodemographic measure with more than 2-factor levels (ie, level of education and subjective social status), separate one-way ANOVAs were conducted. These included the sociodemographic measures as predictors and the attitudes toward psychotherapy scales as the dependent variables. Effect sizes for the ANOVAs are reported as η^2^, with the values η^2^=.01, η^2^=.06, and η^2^=.14 corresponding to small, medium, and large effects, respectively [62]. Significant main effects in the ANOVAs were followed up with Bonferroni-corrected pairwise *t* tests. Uncorrected *P* values and corrected α-thresholds are reported for these post hoc *t* tests. Participants could opt out of any question, resulting in a missing value for that item. Individuals with missing data for one item were excluded from all analyses including this item, for example, a scale or mean sum score was not calculated for an individual if a specific item relevant to that scale or mean sum score was missing, but it was calculated for other scales of the same questionnaire that do not contain that item if all other items were complete.

## Results

### Sample Characteristics and Descriptive Statistics

A sample of 2000 individuals was recruited. The sample characteristics can be found in Table 1 (cumulative percentages may not add up to precisely 100% and sample sizes may vary due to weighting and rounding). Descriptive statistics for the QAPT, HLS-EU-Q16, and MHL-W-G questionnaires across the entire sample are in Table 2. Detailed descriptive statistics for the QAPT attitudes and nonacceptance subscales are in Tables 3 and 4, respectively, and detailed descriptive statistics for each QAPT item can be found in Multimedia Appendix 1.

**Table 1:** Characteristics of the weighted and unweighted sample.

Variable			Unweighted sample (n=2000), n (%)		Weighted sample (n=2000), n (%)
**Gender**					
	Men	957 (47.9)		980 (49.0)	
	Women	1043 (52.1)		1020 (51.0)	
**Age group (years)**					
	16-29	461 (23.1)		355 (17.8)	
	30-45	433 (21.6)		475 (23.7)	
	46-64	599 (29.9)		655 (32.7)	
	>65	507 (25.4)		515 (25.8)	
**Level of education**					
	Low	349 (17.4)		349 (17.5)	
	Middle	847 (42.4)		861 (43.0)	
	High	792 (39.6)		782 (39.1)	
	Missing	12 (0.6)		8 (0.4)	
**Social status**					
	Low	312 (15.6)		315 (15.7)	
	Middle	1397 (69.8)		1389 (69.4)	
	High	291 (14.5)		296 (14.8)	
**Migration background**					
	No	1871 (93.5)		1876 (93.8)	
	Yes	129 (6.5)		124 (6.2)	
**Own experience with psychotherapy**					
	No	1376 (68.8)		1379 (68.9)	
	Yes	603 (30.1)		596 (29.8)	
	Missing	21 (1.1)		25 (1.2)	
**Family experience with psychotherapy**					
	No	1062 (53.1)		1053 (52.6)	
	Yes	870 (43.5)		879 (43.9)	
	Missing	68 (3.4)		68 (3.4)	
**General health literacy categories**					
	Inadequate	192 (9.6)		190 (9.5)	
	Problematic	540 (27.0)		545 (27.3)	
	Adequate	1255 (62.7)		1252 (62.6)	
	Missing	13 (0.7)		13 (0.6)	

**Table 2:** Descriptive statistics of the QAPT^a^, HLS-EU-Q16^b^, and MHL-W-G^c^ questionnaires.

Variable			Sample, n		Score, mean (SD)		Score, 95% CI of the mean	Score, median (range)
**QAPT**								
	Positive attitudes toward psychotherapy	1994		3.28 (0.54)		3.26-3.31		3.33 (1-4)
	Nonacceptance of society	1996		2.06 (0.61)		2.03-2.08		2.00 (1-4)
**HLS-EU-Q16**			1987		12.92 (3.03)		12.79-13.05	14.00 (0-16)
**MHL-W-G**			1994		52.03 (11.02)		51.55-52.51	52.00 (16-80)

^a^QAPT: Questionnaire on Attitudes Towards Psychotherapeutic Treatment.

^b^HLS-EU-Q16: European Health Literacy Survey instrument.

^c^MHL-W-G: Mental Health Literacy Tool for the Workplace (German version).

**Table 3:** Descriptive statistics for the QAPT^a^ attitudes subscale across the sociodemographic groups.

Variable			Score, mean (SD)		Score, 95% CI of the mean		Score, median
**Gender**							
	Men	3.24 (0.55)		3.20-3.27		3.33	
	Women	3.33 (0.52)		3.29-3.36		3.33	
**Level of education**							
	Low	3.20 (0.56)		3.14-3.25		3.17	
	Middle	3.23 (0.54)		3.19-3.27		3.17	
	High	3.38 (0.50)		3.25-3.42		3.50	
**Social status**							
	Low	3.22 (0.55)		3.16-3.28		3.17	
	Middle	3.28 (0.52)		3.25-3.31		3.33	
	High	3.35 (0.57)		3.30-3.43		3.50	
**Migration background**							
	No	3.29 (0.54)		3.25-3.31		3.33	
	Yes	3.25 (0.53)		3.15-3.34		3.33	
**Own experience with psychotherapy**							
	No	3.21 (0.54)		3.18-3.23		3.17	
	Yes	3.46 (0.48)		3.42-3.50		3.50	
**Family experience with psychotherapy**							
	No	3.22 (0.56)		3.19-3.25		3.17	
	Yes	3.36 (0.50)		3.32-3.39		3.33	

^a^QAPT: Questionnaire on Attitudes Towards Psychotherapeutic Treatment.

**Table 4:** Descriptive statistics for the QAPT^a^ nonacceptance subscale across the sociodemographic groups.

Variable			Score, mean (SD)		Score, 95% CI of the mean		Score, median
**Gender**							
	Men	2.13 (0.60)		2.09-2.17		2.20	
	Women	1.99 (0.61)		1.95-2.02		2.00	
**Level of education**							
	Low	2.05 (0.61)		1.99-2.12		2.00	
	Middle	2.05 (0.60)		2.01-2.09		2.00	
	High	2.06 (0.61)		2.02-2.10		2.00	
**Social status**							
	Low	2.16 (0.59)		2.09-2.22		2.20	
	Middle	2.05 (0.60)		2.02-2.09		2.00	
	High	1.96 (0.63)		1.89-2.04		1.80	
**Migration background**							
	No	2.06 (0.60)		2.03-2.08		2.00	
	Yes	2.07 (0.64)		1.96-2.19		2.00	
**Own experience with psychotherapy**							
	No	2.08 (0.59)		2.05-2.11		2.00	
	Yes	1.99 (0.64)		1.94-2.04		2.00	
**Family experience with psychotherapy**							
	No	2.12 (0.60)		2.08-2.15		2.20	
	Yes	1.98 (0.61)		1.94-2.02		2.00	

^a^QAPT: Questionnaire on Attitudes Towards Psychotherapeutic Treatment.

### Reliability Analyses

The reliability analyses revealed an internal consistency of α=.83 (good) for the attitudes subscale and α=.74 (acceptable) for the nonacceptance subscale of the QAPT. The internal consistency was α=.80 (good) for the HLS-EU-Q16 and α=.92 (excellent) for the MHL-W-G.

### Attitudes Toward Psychotherapy and General and Mental Health Literacy

The attitudes subscale of the QAPT correlated negatively with the nonacceptance subscale questionnaire (*r*=−0.17, 95% CI −0.21 to −0.13; *P*<.001). General health literacy correlated positively with the attitudes subscale (*r*=0.14, 95% CI 0.10-0.18; *P*<.001) and negatively with the nonacceptance subscale (*r*=−0.32, 95% CI −0.36 to −0.28; *P*<.001). Mental health literacy correlated positively with the attitudes subscale (*r*=0.18, 95% CI 0.13-0.22; *P*<.001) and negatively with the nonacceptance subscale (*r*=−0.23, 95% CI −0.27 to −0.19; *P*<.001).

### Attitudes Toward Psychotherapy and Experience with Psychotherapy

Participants who have received treatment for a mental disorder reported significantly more positive attitudes toward psychotherapy than participants without treatment experience (t_1260.12_=−10.40, *P*<.001, Cohen *d*=−0.49). Likewise, participants who have received treatment for a mental disorder reported significantly less nonacceptance regarding psychotherapy than participants without treatment experience (t_1050.95_=3.06, *P*=.002, Cohen *d*=0.16).

Similarly, participants who have a close family member who received treatment for a mental disorder reported significantly more positive attitudes toward psychotherapy than participants without family treatment experience (t_1912.06_=−5.66, *P*<.001, Cohen *d*=−0.26). Likewise, participants who have a close family member who received treatment for a mental disorder reported significantly less nonacceptance regarding psychotherapy than participants without family treatment experience (t_1926_=4.77, *P*<.001, Cohen *d*=0.22).

### Attitudes Toward Psychotherapy and Sociodemographic Measures

Age correlated negatively with the attitudes subscale (*r*=−0.11, 95% CI −0.16 to −0.07, *P*<.001) and negatively with the nonacceptance subscale (*r*=−0.08, 95% CI −0.12 to −0.03, *P*<.001).

There were significant gender differences for both subscales. Women reported higher positive attitudes toward psychotherapy than men (t_1992_=−3.60, *P*<.001, Cohen *d*=−0.16). Women also reported lower nonacceptance than men (t_1994_=5.29, *P*<.001, Cohen *d*=0.24).

Subjective social status had a significant effect on both the attitude subscale (*F*_2,1991_=5.25, *P*=.005, η^2^=.005) and the nonacceptance subscale (*F*_2,1993_=7.71, *P*<.001, η^2^=.008). Post hoc Bonferroni-corrected *t* tests for the attitudes subscale (Bonferroni-corrected α=.017) revealed significant differences between the low and high subjective social status groups (t_607_=−3.08, *P*=.002, Cohen *d*=−0.25). Differences between the middle and high (t_1678_=−2.34, *P*=.02, Cohen *d*=−0.15) and the low and middle subjective social status groups (t_1698_=−1.82, *P*=.07, Cohen *d*=−0.11) were not significant after Bonferroni correction. Descriptively, positive attitudes were lowest in individuals with low subjective social status, followed by middle and high subjective social status. Post hoc Bonferroni-corrected *t* tests for the nonacceptance subscale (Bonferroni-corrected α=.017) revealed significant differences between the low and middle (t_1699_=2.72, *P*=.007, Cohen *d*=0.17) and low and high subjective social status groups (t_608_=3.88, *P*<.001, Cohen *d*=0.32). The difference between the middle and high subjective social status groups was not significant after Bonferroni correction (t_1679_=2.32, *P*=.02, Cohen *d*=0.15). Descriptively, nonacceptance was highest in individuals with low subjective social status, followed by middle and high subjective social status.

Level of education had a significant effect on the attitudes subscale (*F*_2,1983_=22.27, *P*<.001, η^2^=.021). Post hoc Bonferroni-corrected *t* tests for the attitudes subscale (Bonferroni-corrected α=.017) revealed significant differences between the low and high educational levels (t_1126_=−5.52, *P*<.001, Cohen *d*=−0.36) and the middle and high educational levels (t_1635.23_=−5.82, *P*<.001, Cohen *d*=−0.29), but not between the low and middle educational levels (t_1204_=−1.01, *P*=.32, Cohen *d*=−0.06). Descriptively, positive attitudes were highest in individuals with high educational levels, followed by middle and low educational levels.

There was no significant effect of level of education on the nonacceptance subscale (*F*_2,1984_=0.02, *P*=.99, η^2^<.001). Descriptively, participants with low, middle, and high levels of education reached similar scores.

There were no significant differences for the attitude subscale between individuals with and without a migration background (t_1992_=0.73, *P*=.46, Cohen *d*=0.07). Likewise, there were no significant differences for the nonacceptance subscale between individuals with and without a migration background (t_1994_=−0.29, *P*=.77, Cohen *d*=−0.03).

Results of the inferential analyses for the unweighted dataset can be found in the Multimedia Appendix 1. Importantly, weighting had no effect on the inferential conclusions except for one analysis. The post hoc *t* test for the difference between the low and high subjective social status groups did not reach significance in the unweighted dataset after the Bonferroni correction.

## Discussion

### Overview

This study investigated the associations between attitudes toward psychotherapeutic treatment and both general and mental health literacy in a large and representative sample of the general population in Germany. In addition, attitudes toward psychotherapeutic treatment were analyzed concerning interindividual differences in terms of experience with mental health treatment and sociodemographic factors.

### Principal Findings

Overall, participants expressed largely positive attitudes toward psychotherapeutic treatment and reported relatively low perceived nonacceptance. However, significant interindividual differences were observed and are discussed below.

Importantly, while our results demonstrate several statistically significant results, the practical significance of these findings has to be considered before interpretation. All effect sizes (Cohen *d* and η^2^) reported here fall into the small to medium range [61,62]. In addition, some of the CI of the means overlap. This means that the practical impact of our findings in real-world settings may be limited. Nonetheless, these findings point to potentially vulnerable sociodemographic groups in need of specific interventions to promote positive attitudes toward psychotherapy. The small effect sizes observed here suggest that while tailored interventions for specific groups may be more effective due to a better fit between the specific needs of a sociodemographic group and the intervention, there is also a potential overall benefit of broader general-population interventions [63,64].

The questionnaire scales used in this study reached acceptable to excellent internal consistency coefficients speaking for a high degree of interrelatedness of the scale items and their overall reliability which is an important prerequisite for the validity of the results [60]. Furthermore, weighting did not have a marked effect on the conclusions drawn from the statistical findings which highlights the generalizability of the findings.

### General and Mental Health Literacy

Both higher general and higher mental health literacy were associated with more positive attitudes and lower nonacceptance of psychotherapeutic treatment. For mental health literacy, this finding aligns well with previous research [12,32,33,35]. Of note, these previous studies mostly used the simple identification of vignettes as a measure of mental health literacy. Here, we used a much broader instrument for mental health literacy more closely mirroring the definition of mental health literacy by also covering aspects of support, prevention, and use of resources [28,53]. For the first time, we show here that by including these broader aspects of mental health literacy positive associations with positive attitudes and negative associations with nonacceptance can be obtained, significantly extending previous observations in studies using vignette identification as a proxy for mental health literacy. Another novel contribution of our investigation is that we replicated initial findings of the associations between the QAPT and MHL-W-G scales previously observed only in a small sample of working individuals in a substantially larger general population sample with similar effect sizes [43]. Notably, here, we also add to the literature by showing a positive association between general health literacy and the attitudes measure, which to our knowledge was previously only reported in one study [34]. Here, we replicate these initial findings in a larger sample and generalize them to another measure of general health literacy, namely the widely used HLS-EU-Q16. This suggests that individuals with higher levels of both general and mental health literacy are more likely to exhibit positive attitudes toward psychotherapy and perceive societal acceptance to be higher. Consequently, they might also be more likely to seek treatment when faced with mental problems [19]. Crucially, however, it has to be noted that our cross-sectional study design does not allow to infer causal associations between the investigated constructs (also refer to limitations below; [65]). Therefore, we cannot unequivocally conclude whether inadequate or problematic health literacy is indeed the antecedent and origin of less positive attitudes toward psychotherapy. We recommend future studies with appropriate study designs to test for causal effects, for example, randomized controlled trials. In addition, other potentially confounding variables should be carefully assessed and controlled for.

The present findings should also be interpreted in the context of mental health stigma in general. Although some progress in reducing stigma has been made in the past years substantial stigma against people with mental health problems remains [13,14]. Crucially, individuals with more stigmatizing attitudes show less active help-seeking behavior [17]. The promotion of health literacy with targeted interventions along with far-reaching antistigma campaigns could therefore also lead to higher rates of individuals seeking treatment [66]. Importantly, antistigma interventions may be more effective when focusing on psychosocial instead of biogenetic causes for mental disorders [35]. In addition to these interventions focusing on individual barriers, structural barriers to psychotherapeutic treatment (although not the focus of the present research) should not be disregarded and must be addressed as well. For example, waiting periods should be significantly reduced and provision should be increased, especially in rural areas [2,6,7].

### Experience with Psychotherapy

Attitudes were more positive, and nonacceptance was lower in participants who have received treatment for mental disorders in the past or those who have a close relative with treatment experience. This is consistent with previous research [8,35]. It is noteworthy, that the treatment experience of a close family member may already improve the treatment attitudes of their relatives although with lower effect sizes than personal experience. Speaking openly about mental health treatment experiences and thus providing information about treatments might therefore be a valuable path to lowering psychotherapy stigma [49]. However, as no causal conclusions can be drawn from our study design, it is possible that positive treatment attitudes are not the result of personal or family treatment experience but that those with a priori more positive treatment attitudes are more likely to seek treatment.

### Sociodemographic Factors

Women exhibited significantly more positive attitudes and reported lower perceived societal nonacceptance (ie, higher acceptance) of psychotherapeutic treatment compared to men. This finding aligns with previous research [10,67] and may be attributed to masculinity ideals and self-stigma, preventing men from seeking help for mental health problems [68,69].

Regarding age, more positive attitudes toward psychotherapeutic treatment were found in younger individuals, consistent with some [8] but not all previous investigations [10,70]. However, surprisingly, younger individuals also displayed higher levels of nonacceptance compared to older individuals, suggesting a discrepancy between perceived societal acceptance and individual attitudes. This pattern of results might be interpreted as higher awareness of the persisting mental health stigma in the community in younger individuals, although a generational shift toward less stigma is slowly taking place [14]. Notably, in our data, correlations with age were relatively small for both subscales. Integrating our results with previous literature, an overall heterogeneous picture emerges [8,10,70]: age seems to have no or a very small effect on attitudes toward psychotherapy and how it is perceived to be accepted by society. Future interventions to improve help-seeking for mental problems should therefore address all age groups.

Attitudes toward psychotherapy were also related to the subjective social status of the participants. Specifically, positive attitudes increased, and nonacceptance decreased with higher subjective social status, although the post hoc test between the high and low social status groups for the attitudes subscale narrowly missed significance in the unweighted dataset (refer to Multimedia Appendix 1). Social status has been identified as a major determinant of help-seeking behavior in the past [71,72]. However, more research is needed to determine the role of social status in attitudes toward psychotherapy. Critically, social status also plays a role in access to psychotherapy. Individuals with lower social status are less likely to be offered treatment than individuals with higher social status suggesting that structural barriers are also higher for these individuals than those with higher social status [73]. Potential financial barriers to access to psychotherapeutic treatment should also be considered but do not apply to the majority of our participants as the German public health insurance that insures about 90% of the population covers psychotherapeutic treatment for many diagnoses [74-76]. Treatment attitudes and treatment seeking should be carefully addressed in help-seeking interventions for individuals with lower social status and treatment should be provided equally to individuals of all social status groups. As general and mental health literacy were found to be low in individuals with lower subjective social status, these groups might particularly benefit from interventions [31,77]. There is first evidence to suggest that individuals with low social status might particularly benefit from culturally appropriate and tailored interventions that are based on scientific theories and incorporate different modalities, for example, using print materials and offering direct contact with the interventionists [63,64,78].

Higher educational levels were associated with more positive attitudes toward psychotherapy. However, there was no association between education and perceived acceptance of psychotherapy. In the literature, there is heterogeneous evidence concerning the role of education in attitudes toward mental health help-seeking [8,10,53]. Therefore, more research in this area is recommended to determine the role of education in help-seeking attitudes. Our results carefully suggest that programs directed at individuals with lower levels of education could be particularly beneficial.

Migration background was not related to neither positive attitudes nor perceived nonacceptance in our sample. This result is surprising given previous evidence of more negative attitudes toward psychotherapeutic treatment, and simultaneous higher endorsement of pharmacotherapy, in people with a migration background [44-46]. However, as we only included individuals with sufficient knowledge of the German language, it cannot be ruled out that our sample of individuals with a migration background was not representative of all individuals with a migration background in Germany (refer to *Limitations* section). Future studies could try to overcome this problem by providing translated questionnaires to reach a broader segment of the population with a migration background. In addition, substantial cultural differences have been delineated in the past, which is why we recommend investigating more thoroughly how the cultural backgrounds of the individuals with migration backgrounds are associated with their endorsement of psychotherapeutic treatment [44,79,80], but our data does not allow more fine-grained analyses. Notably, however, interventions to increase the use of mental health care may be more effective when tailored to the specific needs of minority groups and different cultures [47,63,64,78].

Overall, the findings presented here imply that more interventions to improve attitudes toward psychotherapy might be necessary and might be achieved by explicitly targeting both general and health literacy in vulnerable segments of the community. Our findings suggest that male individuals, those with higher age, lower social status, and lower levels of education might particularly benefit from interventions to promote positive attitudes toward psychotherapy. Of note, while a large number of interventions have already been developed and evaluated in the past, systematic reviews of the literature show substantial heterogeneity in the efficacy of these interventions and call for more high-quality evaluations [36-39]. In particular, more effort needs to be put into interventions in different cultures and across ethnic minority groups [40,41,80]. Notably, there are first promising results, for example concerning digital mental health interventions that are both cost-effective and easy to implement [39]. Web-based interventions appear to be generally efficacious when they include certain active components such as interactivity, target-group specificity, evidence-based content, and a sequential modular concept [81], for example as part of an e-learning platform [82,83]. While potential improvements in health literacy may not directly translate to increased help-seeking behavior, further research is needed to explore this connection [81].

### Limitations

The findings presented here have to be interpreted in the light of some limitations. First, both attitudes and health literacy were assessed via self-reports. Future studies could focus on more applied measures rather than self-reports for a broader scope and higher ecological validity. Second, 6.2% of the participants in the weighted sample reported having a migration background. This is substantially lower than recent census data from Germany [84], suggesting that our sample may not be representative in terms of the proportion of individuals with a migration background. Third, giving our participants the option to opt-out from specific items might have resulted in systematically missing data which could have affected the generalizability of the results. However, the number of individuals using that option was relatively low, with the highest number (3.4%) for the item asking about family experience with psychotherapy which we speculate might have been the result of lack of knowledge. Importantly, the proportions of missing values were smaller for the main questionnaires used here (eg, 0.3% and 0.2% for the QAPT scales, refer to Table 2). As these proportions are substantially lower than the recommended threshold of 5%, no imputation analyses for missing values were conducted and all analyses were conducted with all available data [85]. Fourth, we relied on data collected on the web which might have resulted in lower data quality [86,87]. However, with this interview mode, a substantially larger sample size could be achieved, simultaneously reducing error variance. In addition, by working with the forsa.omninet panel a few issues common to web-based surveys could be mitigated. For example, participants were randomly selected and could not apply to take part in the study, people who rarely use the internet were also represented due to the panel recruitment via telephone, and multiple registrations of the same individuals were prevented. However, the sample is only representative of the population with internet access as data collection took place exclusively on the internet, which is why results may not be generalizable to individuals without internet access. Finally, as noted above, the present investigation used a cross-sectional study design. Consequently, the results cannot be interpreted in terms of causal relationships [65], which is why further research is needed with more sophisticated study designs including longitudinal and experimental studies that control for potentially confounding factors.

### Summary and Conclusion

In this study, positive attitudes toward psychotherapy and perceived societal acceptance of psychotherapeutic treatment were investigated in a large general population sample in Germany. Our results highlight a positive association between these factors and both general and mental health literacy. Individuals who have been treated for mental problems or who have relatives with treatment experience reported more favorable attitudes and higher perceived acceptance of society than those without treatment experience. In terms of sociodemographic differences, it was shown that being a woman, younger age, higher social status, and higher levels of education were associated with more positive attitudes toward psychotherapeutic treatment. Being a woman, older age, and higher subjective social status were associated with higher perceived societal acceptance of psychotherapy. Since previous studies suggest that target group-specific interventions have the potential to improve health literacy, future studies should focus on establishing whether tailored interventions designed to increase general or mental health literacy and aimed at individuals with deficits in this area may improve attitudes toward seeking help for mental problems.

## Appendix

Multimedia Appendix 1 Supplementary analyses and tables.

## Abbreviations

HLS-EU-Q16: European Health Literacy Survey instrument
MHL-W-G: Mental Health Literacy Tool for the Workplace (German version)
QAPT: Questionnaire on Attitudes Towards Psychotherapeutic Treatment

## Data Availability

The datasets generated and analyzed during this study are available from the Stiftung Gesundheitswissen on reasonable request.
