# Supplementary material for: Attitudes Toward Psychotherapeutic Treatment and Health Literacy in a Large Sample of the General Population in Germany: Cross-Sectional Study
Source: JMIR Public Health Surveill. 2025 Jan 24;11:e67078. doi: 10.2196/67078 (PMC11806273; doi:10.2196/67078)
Supplement: Multimedia Appendix 1 [file publichealth_v11i1e67078_app1.pdf]

Supplementary Material

Results for the unweighted sample

Reliability analyses

The reliability analyses revealed an internal consistency of $\alpha = .84$ for the attitudes subscale and $\alpha = .74$ for the non-acceptance subscale of the QAPT. The internal consistency was $\alpha = .80$ for the HLS-EU-Q16 and $\alpha = .91$ for the MHL-W-G.

Attitudes towards psychotherapy and general and mental health literacy

The attitudes subscale of the QAPT correlated negatively with the acceptance subscale questionnaire ($r = -0.19$, $P < .001$). General health literacy correlated positively with the attitudes subscale ($r = 0.13$, $P < .001$) and negatively with the non-acceptance subscale ($r = -0.32$, $P < .001$). Mental health literacy correlated positively with the attitudes subscale ($r = 0.17$, $P < .001$) and negatively with the non-acceptance subscale ($r = -0.23$, $P < .001$).

Attitudes towards psychotherapy and experience with psychotherapy

Participants who have received treatment for a mental disorder reported significantly more positive attitudes towards psychotherapy than participants without treatment experience, $t(1267.31) = -9.84$, $P < .001$, $d = -0.46$. Likewise, participants who have received treatment for a mental disorder reported significantly less non-acceptance regarding psychotherapy than participants without treatment experience, $t(1073.82) = 3.33$, $P < .001$, $d = 0.17$.

Similarly, participants who have a close family member who received treatment for a mental disorder reported significantly more positive attitudes towards psychotherapy than participants without family treatment experience, $t(1910.48) = -6.26$, $P < .001$, $d = -0.28$. Likewise, participants who have a close family member who received treatment for a mental disorder reported significantly less non-acceptance regarding psychotherapy than participants without family treatment experience, $t(1926) = 4.65$, $P < .001$, $d = 0.21$.

Attitudes towards psychotherapy and sociodemographic measures

Age correlated negatively with the attitudes subscale ($r = -0.12$, $P < .001$) and negatively with the non-acceptance subscale ($r = -0.08$, $P < .001$).

There were significant gender differences for both subscales. Women reported higher positive attitudes towards psychotherapy than men $t(1992) = -4.09$, $P < .001$, $d = -0.18$). Women also reported lower non-acceptance than men, ($t(1994) = 5.20$, $P < .001$, $d = 0.23$).

Subjective social status had a significant effect on both the attitude subscales ($F(2,1991) = 3.01$, $P = .049$, $\eta^2 = .003$) and the non-acceptance subscale ($F(2,1993) = 7.62$, $P < .001$, $\eta^2 = .008$). Post-hoc Bonferroni-corrected t-tests for the attitudes subscale (Bonferroni-corrected $\alpha = .017$) revealed no significant differences between the low and high subjective social status groups, $t(599) = -2.30$, $P = .022$, $d = -0.18$. Differences between the middle and high ($t(1680) = -1.47$, $P = .141$, $d = -0.10$) and the low and middle subjective social status groups ($t(1703) = -1.71$, $P = .088$, $d = -0.11$) were also not significant after Bonferroni correction. Post-hoc Bonferroni-corrected t-tests for the non-

acceptance subscale revealed significant differences between the low and middle (t(1704) = 3.10, P = .002, d = 0.19) and low and high subjective social status groups (t(600) = 3.74, P < .001, d = 0.31). The difference between the middle and high subjective social status groups was not significant after Bonferroni correction, t(1682) = 1.80, P = .073, d = 0.12.

Level of education had a significant effect on the attitudes subscale (F(2,1979) = 28.12, P < .001, η^2 = .028). Post-hoc Bonferroni-corrected t-tests for the attitudes subscale (Bonferroni-corrected α = .017) revealed significant differences between the low and high educational levels (t(597.87) = −5.91, P < .001, d = −0.40) and the middle and high educational levels (t(1630.89) = −6.58, P < .001, d = −0.33), but not between the low and middle educational levels (t(1190) = −1.04, P = .297, d = −0.07).

There was no significant effect of level of education on the non-acceptance subscale, F(2,1981) = 0.01, P = .995, η^2 < .001.

There were no significant differences for the attitude subscale between individuals with and without a migration background t(1992) = 0.51, P = .612, d = 0.05. Likewise, there were no significant differences for the non-acceptance subscale between individuals with and without a migration background, t(1994) = −1.00, P = .318, d = −0.09.

Table S1. Descriptive unweighted statistics of the QAPT, HLS-EU-Q16 and MHL-W-G questionnaires.

**Variable**	***N***	***Mean***	***95%-confidence interval of the mean***	***Median***	***Standard deviation***	***Range***
**QAPT**						
Positive attitudes towards psychotherapy	1994	3.28	[3.26; 3.31]	3.33	0.54	1–4
Non-acceptance of society	1996	2.06	[2.03; 2.08]	2.00	0.61	1–4
**HLS-EU-Q16**	1987	12.91	[12.78; 13.04]	14.00	3.04	0–16
**MHL-W-G**	1994	52.05	[51.57; 52.53]	52.00	10.92	16–80

Individual item responses for the QAPT questionnaire

Table S2. Individual item responses to the QAPT items in the weighted sample.

Item No.	Do not agree	Somewhat do not agree	Somewhat agree	Agree	Missing
**1**	57 (2.9%)	201 (10.0%)	885 (44.3%)	855 (42.8%)	1 (0.1%)
**2**	780 (39.0%)	779 (38.9%)	343 (17.2%)	97 (4.8%)	1 (0.1%)
**3**	86 (4.3%)	239 (11.9%)	856 (42.8%)	817 (40.8%)	4 (0.2%)
**4**	64 (3.2%)	263 (13.1%)	1010 (50.5%)	659 (32.9%)	4 (0.2%)
**5**	356 (17.8%)	643 (32.1%)	739 (37.0%)	260 (13.0%)	2 (0.1%)
**6**	449 (22.4%)	684 (34.2%)	664 (33.2%)	202 (10.1%)	1 (0.0%)
**7**	968 (48.4%)	796 (39.8%)	188 (9.4%)	45 (2.3%)	2 (0.1%)
**8**	27 (1.3%)	96 (4.8%)	925 (46.3%)	950 (47.5%)	2 (0.1%)
**9**	37 (1.8%)	98 (4.9%)	784 (39.2%)	1079 (54.0%)	2 (0.1%)
**10**	111 (5.6%)	410 (20.5%)	827 (41.3%)	651 (32.5%)	1 (0.1%)
**11**	40 (2.0%)	186 (9.3%)	1040 (52.0%)	732 (36.6%)	2 (0.1%)

Note: QAPT: Questionnaire on Attitudes towards Psychotherapeutic Treatment [1,2,3]. The items were originally presented in German. Item 10 was reversed for further analyses, but responses in the table are presented in their original form.

References

[1] Schulz W, Shin MA, Schmid-Ott G. Einstellungen gegenüber Psychotherapie in Südkorea und Deutschland : Eine kulturvergleichende Studie. [Attitudes towards psychotherapy in South Korea and Germany : A cross-cultural comparative study]. Nervenarzt 2018;89(1):51-57. PMID:27909737

[2] Schmid-Ott G, Reibold S, Ernst G, Niederauer HH, Künsebeck H-W, Schulz W, Lamprecht F, Jäger B. Development of a Questionnaire to Assess Attitudes towards Psychotherapeutic Treatment. Dermatol Psychosom 2003;4(4):187-193. doi:10.1159/000075905

[3] Ditte D, Schulz W, Schmid-Ott G. Einstellung gegenüber der Psychotherapie in der russischen Bevölkerung und in der Bevölkerung mit einem russischen/sowjetischen kulturellen Hintergrund in Deutschland. Eine Pilotstudie. [Attitude towards psychotherapy in the Russian population and in the population with a Russian/Soviet cultural background in Germany. A pilot study]. Nervenarzt 2006;77(1):64-72. PMID:15776258
